# Editorial: Advances in understanding the mechanisms of pulmonary hypertension

**DOI:** 10.3389/fcvm.2023.1249889

**Published:** 2023-09-15

**Authors:** Ashfaq Ahmad, Peng Zhang, Lingling Li, Xiaoyu Wang, Ahmed Ali Mohsen, Yousen Wang, Fenling Fan

**Affiliations:** Department of Cardiovascular Medicine, First Affiliated Hospital, Xi'an Jiaotong University, Xi'an, China

**Keywords:** pulmonary hypertension, idiopathic pulmonary hypertension, pulmonary vascular remodeling, heart failure, cardiovascular diseae

**Editorial on the Research Topic** Advances in understanding the mechanisms of pulmonary hypertension

Pulmonary hypertension (PH) is a vascular disorder characterized by elevated pulmonary arterial pressure (resting mean pulmonary arterial pressure >20 mmHg) and pulmonary vascular remodeling, that eventually leads to a high rate of morbidity and mortality due to right heart failure (1). With increased knowledge of PH, the most recent guideline has gained much attention as the reasons of below: (1) PH has been a common cardiovascular disease with an estimated prevalence of about 1% globally and approximately 10% in individuals aged above 65 years (1). (2) PH remains a lethal disease with one-year mortality up to 20% in the intermediate-risk patients and over 20% in the high-risk group, according to registry data quoted in the 2022 ESC/ERS guideline (1). (3) The etiology and mechanism of PH are complicated and are still not fully clear, though there are 5 categories recognized according to the underlying causes. These are pulmonary arterial hypertension (PAH, Group 1), pulmonary hypertension due to left heart or lung/hypoxia diseases (Group 2 and Group 3 respectively), PH due to pulmonary arterial obstruction (Group 4), and PH due to miscellaneous causes (Group 5). These 5 groups may be very different in specific therapies as pathogenesis and mechanism are basically distinct amongst them. Therefore, unraveling the underlying mechanisms in different groups of PH is a topical field of research, as exemplified by the studies reported in this special issue “Advances in understanding the mechanisms of pulmonary hypertension”.

Previously, most target drugs, such as Bosentan, Ambrisentan, etc. are approved on the basis of improvement in the 6-Minute Walk Distance (6MWD) as the primary endpoint. Subsequently, a composite endpoint (including death, atrial septostomy, lung transplantation, initiation of treatment with intravenous or subcutaneous prostanoids, or worsening of PAH, etc.) was the primary endpoint in SERAPHIN and GRIPHON studies, on which for approval of Macitentan and Selexipag use as PAH specific medications were based (2, 3). A study from An Wang et al. in this issue specifically compared Clinical Worsening (TTCW) with 6MWD and concluded that TTCW would provide a better assessment for PAH medication trials. These changes in the primary endpoint of clinical trials give a clue, in conjunction with previously mentioned observations, that PAH not only damages pulmonary vessels but also right heart leading to a high rate of death. The fundamental mechanisms in the various subtypes are likely different so further investigations are definitely needed.

One of the more aggressive subtypes of PAH is idiopathic PAH (IPAH) characterized by hemodynamic changes of PAH but not associated with another disease (4). IPAH is life-threatening and the most difficult subtype to diagnose and treat. As all the other reasons for PAH have to be excluded before reaching a diagnosis of IPAH, the key pathological change of IPAH is pulmonary vascular remodeling beyond arterial constriction. Identifying the underlying mechanism for IPAH and developing therapy targeting vascular remodeling remains challenging. In recent years, inflammation has been revealed as an important factor in the development of PAH. The lung tissues of IPAH individuals are overrun by immune cells such as lymphocytes, macrophages, and mast cells, as determined by histologic examinations (5). Moreover, interleukin (IL)-1β and tumor necrosis factor (TNF)-α, circulating inflammatory cytokines, were found to be significantly increased in blood from patients with IPAH (6). The immune response is intimately tied to peripheral blood mononuclear cells (PBMCs), which are composed of lymphocytes, monocytes, and other cell types. The studies suggested that PBMCs were significantly associated with the development of IPAH (7). A study in this issue by Chen et al. aimed to build networks of genes co-expressed in patients with IPAH, and assess immune-related differentially expressed genes (IRDEGs) via bioinformatics to explore the potential mechanism underlying IPAH which might provide novel biomarkers and therapeutic targets for IPAH. They performed enrichment analysis, and examined immune cell infiltration in lung tissues from IPAH patients. They found a significant association between inflammatory response, increment in cytosolic calcium ion, and metabolic dysregulation in IPAH. Also, they found T cells, CD4 memory resting and macrophage M1 significantly infiltrated lung samples from patients with IPAH. Through the analysis of immune-related differentially expressed genes (IRDEGs) of the lung dataset and PBMCs dataset, C-X-C motif chemokine ligand 10 (CXCL10) and vasoactive intestinal peptide receptor 1(VIPR1) were identified as hub genes. In order to further investigate the underlying mechanism of PAH, another finding from Yang et al. in this issue showed on gene hub EP300, an acetyltransferase, played diverse roles in cell proliferation, differentiation, and apoptosis. This finding, along with previous studies on the involvement of histone acetylase (HDAC) in PAH development indicate HDAC inhibitors have therapeutic potential for the treatment of PAH (4, 5, 8).The authors stated that EP300 is potential to protect PAH. Similar results were also found from Sacilotto’s et, al. study that EP300 can function as a regulatory factor during vascular endothelial growth factor A-induced angiogenesis (9). In a further attempt at unraveling mechanisms at a molecular level, Yang et al. studied a rare mitochondrial disease (HUPRA syndrome), and reported novel SARS2 compound heterozygous variants c.1205G>A/c.680G>A. Genetic testing demonstrated that the mutation c.680G>A, located in a conserved area, altered the structure of the SerRS protein. That patient had substantial PH and the mutations p.Arg402His and p.Arg227Gln were both categorized as variants of uncertain significance (VUS). The study provides important information in terms of these heterozygous variants, however, understanding the detailed molecular mechanism needs further research.

Pulmonary hypertension due to left heart disease (PH-LHD) is frequently encountered, accounting for 65%–80% of PH cases and is also associated with high mortality. Unfortunately, target drugs approved for PAH have so far not been recommended for PH-LHD, in part since the exact mechanism of PH-LHD is still unclear. Although the available pharmacological therapies for LHD, including diuretics, ACE inhibitors, and beta-blocker have improved outcomes, PH-LHD remains progressive and ultimately fatal. As reviewed in the article from Xiao et al., the etiology of PH-LHD is primarily from a passive increase in pulmonary venous resistance, but which is gradually accompanied by pulmonary arterial damage. Consistent with that, the haemodynamic condition advances from isolated post-capillary PH (IpcPH) into combined post- and pre-capillary PH (CpcPH) based upon pulmonary vascular resistance (PVR <2.0 or ≥2.0 WU) (1). Progress in understanding the mechanism of pulmonary vascular remodeling will be particularly important for exploring possible pulmonary vascular target therapy in CpcPH. Based on knowledge that an imbalance existes in transforming growth factor-β (TGF-β)/Smad signaling pathway contributing to pathogenic vascular remodeling in PAH (10), additional research in recent years has proved that Sotatercept, an activin receptor type IIA-Fc (ActRIIA-Fc) fusion protein, significantly reverses abnormal pulmonary vascular remodeling by rebalancing TGF-β/Smad and exerts anti-inflammatory effects in experimental PAH (11). In this issue, Joshi et al. induced transverse aortic constriction (TAC) in mice to test that ActRIIA-Fc improved pulmonary vascular remodeling and alleviated PH from LHD. Their results indicated that ActRIIA-Fc exerts protective cardiopulmonary effects in experimental PH-HFrEF by reducing cardiac remodeling. These findings give new ideas to treating PH-LHD by way of targeting the cardiac remodeling pathway.
